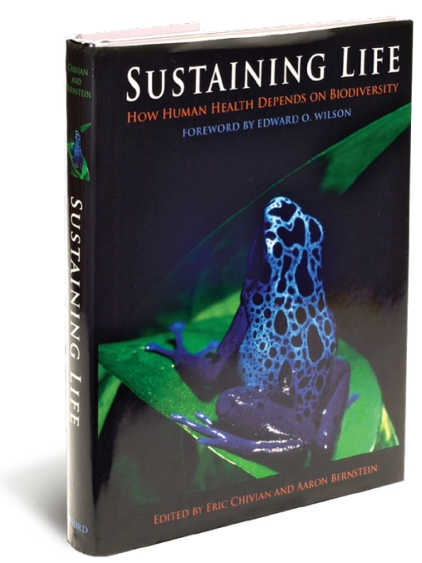# Sustaining Life: How Human Health Depends on Biodiversity

**Published:** 2009-06

**Authors:** Stephen C. Stearns

**Affiliations:** Stephen C. Stearns, professor of ecology and evolutionary biology at Yale University, has been engaged in issues of biodiversity and medicine through his work on extinctions, on the environmental studies major at Yale, and his work on evolutionary medicine

*Edited by Eric Chivian and Aaron Bernstein* New York:Oxford University Press, 2008. 542 pp. ISBN: 978-0-19-517509-7, $34.95

We share this planet with millions of other species, each of which is a unique store of precious information. We are driving those species extinct at 100 to 1,000 times the natural rate—a crime equivalent to tossing books from the Library of Alexandria thoughtlessly into a fire, erasing the shared inheritance of all mankind. To slow or prevent this process, one should surely exhaust all reasonable arguments and actions. This book, a well-edited and beautifully presented multi-author volume, argues for preserving the planet’s biodiversity for practical reasons: Many species benefit humankind through medicine and agriculture.

Assembled by a team of nearly 300 authors and editors, the book starts with overviews of biodiversity and how it is threatened by human activity, claims that biodiversity supports essential ecosystem services, and then introduces medicines from nature. The enlightening and useful sixth chapter reviews the medical value of seven threatened groups—amphibians, bears, primates, gymnosperms, cone snails, sharks, and horseshoe crabs—giving advocates new and valuable ammunition. The next chapter describes how human impacts on ecosystems and their bio-diversity affect the emergence and spread of disease. Two chapters make clear that we rely for healthy food on biodiversity in agriculture. The concluding chapter describes how individuals can conserve biodiversity: reduce consumption and take local action.

The argument is impressive, the production is beautiful, and the cause is just. It is a shame that the logic does not work. Two extremely important elements have been left out: economics and population.

If one chooses the economic strategy, one must be prepared to deal with economic counterarguments. It is not enough to demonstrate that biodiversity has practical value for medicine and agriculture; one must show that it has more such value than other options. If pharmaceutical companies could make money developing drugs via bioprospecting, they would do so; books like this one would not be needed to convince them. And though one chapter argues, convincingly, that biodiversity is higher in organically farmed croplands than in the more intensive alternatives, a trip to the local supermarket, which carries both, reveals that one pays a 30–40% premium for the organic option. Conservation remains a luxury of the rich, not a priority of the poor. The best hope for economic gain from biodiversity lies in genomic surveys of the bacteria and fungi that produce antibiotics.

Human impact on biodiversity equals, roughly, number of humans times the amount consumed per capita, with a correction factor for good or bad behavior. Because solving the population problem is essential to stopping the biodiversity crisis, it is striking that in a long book with a chapter on how we can modify our consumption to protect biodiversity, there is no mention of population. One wonders why, for the logic of population impact is inescapable.

If we could simply give every woman on the planet control over her own reproductive destiny, the mean completed family size might drop naturally, populations would grow less rapidly, and some might even decrease. They would not moderate rapidly enough to prevent many extinctions, but in the long run decreasing the human population of the planet is the only way to stably stop extinctions.

Nor is it particularly credible to advocate reducing consumption when the governments of the world are scrambling frantically to avert a global financial crisis with incentives to increase consumption. Asking individuals to refrain from consuming when most of those around them are trying to consume more—the core of the modern materialist lifestyle—is simply to note the tragedy of the commons without solving it.

Finally, claiming that biodiversity is essential to maintain ecosystem services, without quantifying how many species are actually needed to do it or acknowledging evidence for extensive redundancy in natural ecosystems, risks loss of credibility. Do we really know the minimum number of species that we need? We might lose many of them with no discernible impact on ecosystem services.

This book will certainly help, not hurt, and I am glad it exists, but it will not solve the core problems of economics and population. What we need is the research to discover, and the political courage to implement, effective incentives to reduce global consumption and global birth rates. All else is window dressing.

## Figures and Tables

**Figure:**